# All‐Copper Nanocluster Based Down‐Conversion White Light‐Emitting Devices

**DOI:** 10.1002/advs.201600182

**Published:** 2016-06-21

**Authors:** Zhenguang Wang, Bingkun Chen, Andrei S. Susha, Weihua Wang, Claas J. Reckmeier, Rui Chen, Haizheng Zhong, Andrey L. Rogach

**Affiliations:** ^1^ Department of Physics and Materials Science and Centre for Functional Photonics (CFP)City University of Hong Kong83 Tat Chee AvenueKowloonHong Kong SAR; ^2^ Beijing Key Laboratory of Nanophotonics and Ultrafine Optoelectronic SystemsSchool of Materials Science and EngineeringBeijing Institute of TechnologyBeijing100081China

**Keywords:** aggregation‐induced emission enhancement, copper nanoclusters, down‐conversion light‐emitting devices, photoluminescence, white light

## Abstract

Most of the present‐day down‐conversion white light‐emitting devices (WLEDs) utilize rare‐earth elements, which are expensive and facing the problem of shortage in supply. WLEDs based on the combination of orange and blue emitting copper nanoclusters are introduced, which are easy to produce and low in cost. Orange emitting Cu nanoclusters (NCs) are synthesized using glutathione as both the reduction agent and stabilizer, followed by solvent induced aggregation leading to the emission enhancement. Photoluminescence quantum yields (PL QY) of 24% and 43% in solution and solid state are achieved, respectively. Blue emitting Cu nanoclusters are synthesized by reduction of polyvinylpyrrolidone supported Cu(II) ions using ascorbic acid, followed by surface treatment with sodium citrate which improves both the emission intensity and stability of the clusters, resulting in the PL QY of 14% both in solution and solid state. All‐copper nanocluster based down‐conversion WLEDs are fabricated by integrating powdered orange and blue emitting Cu NC samples on a commercial GaN LED chip providing 370 nm excitation. They show favorable white light characteristics with Commission Internationale de l'Eclairage color coordinates, color rendering index, and correlated color temperature of (0.36, 0.31), 92, and 4163 K, respectively.

## 1 Introduction

Light‐emitting devices (LEDs) have been widely used in displays, automotive headlamps, and traffic signals, with white LEDs (WLEDs) increasingly considered as a useful alternative to replace incandescent light bulbs for general lighting.^1^ The common approach toward white light generation in WLEDs employs single or multiple phosphors which are excited by a blue or near‐UV LEDs chips,^2^ known as a down‐conversion of light. A typical example of this kind of devices is a combination of a yellow‐emitting Ce^3+^‐doped yttrium aluminum garnet (YAG:Ce^3+^) and a blue‐emitting InGaN/GaN diode. Similarly to YAG:Ce^3+^, many of other commercial phosphors rely on rare‐earth elements, such as europium, terbium, and yttrium,^3^ which offer advantages of high stability and photoluminescence (PL) quantum yield (QY). Due to the growing demand, rare‐earth elements are facing a serious shortage of supply, while their recycling procedures may be destructive to the environment.^4^

One deficiency of most WLEDs based on rare‐earth phosphors is their high correlated color temperature (CCT) over 4500 K and rather poor color rendering index (CRI) below 80, which results in a colder white light compared to the incandescent light bulbs.^5^, ^6^ This issue can be overcome by using an additional suitable red emitting rare‐earth phosphor; however, their typically small Stokes shifts lead to a strong reabsorption of emitted light, resulting in the decrease of efficiency and color stability.^5^, ^7^ This triggered an ongoing search for alternative, rare‐earth free phosphors for WLEDs.^8^, ^9^ Among them, metal nanoclusters (NCs)^9^, ^10^ are attractive, as they can be easily synthesized in solution, processed into powders, and, for the noble metal NCs, exhibit strong PL with a large Stokes shift.^11^ Copper is an earth abundant element widely used in different industries, and there have been few reports on down‐conversion WLEDs with Cu NCs as phosphors, mostly in combination with other luminophores.^9^ Yang and co‐workers reported WLEDs employing a combination of blue‐green emitting Cu ribbons (PL QY of 6.5%), yellow emitting Cu nanosheets (PL QY of 3.6%), and red emitting Au nanosheets, self‐assembled from Cu and Au NCs.^9^ Along a similar concept, Liu et al. fabricated WLEDs employing blue‐green emitting Cu sheets (PL QY of 4.6%) and red emitting Au sheets obtained by electrophoretic deposition of Cu and Au NCs.^12^ To the best of our knowledge, there have been no reports on WLEDs where solely Cu NCs as phosphors were employed, eventually due to their relatively low PL QY, which is commonly below 10% for clusters dispersed in solution^12^ and decreases further upon their processing as powders.^13^, ^14^ Emission quenching upon transfer of luminescent nanoparticles from solution to films or powders is a well‐known phenomenon, ascribed to the nonradiative Förster resonant energy transfer of excitons to the neighboring species with lower PL QY.^15^ On the other hand, the pioneering work on the aggregation‐induced emission (AIE) of 1‐methyl‐1,2,3,4,5‐pentaphenylsilole discovered by Tang and co‐workers^16^ opened up new perspectives for designing strongly emitting phosphors, where the issue of aggregation caused quenching is bound to overcome. There have been a number of encouraging reports demonstrating AIE for Cu NCs stabilized with thiol ligands;^17^, ^18^, ^19^, ^20^ as an example, their PL QY could be improved more than tenfold, from 0.45% to 6.6%.^18^ A proper surface treatment of metal NCs is yet another useful strategy to improve their emission intensity both in solution and in the solid state. We recently suggested a postpreparative treatment of Cu NCs with glutathione (GSH), allowing us to improve their PL QY from 8% to 27%.^21^ The bright emission of these Cu NCs supported on polyvinylpyrrolidone (PVP) backbone was fully preserved in the solid state powder, allowing us to employ them as blue emitting component of down‐conversion WLEDs in combination with commercial green and red phosphors.

**Scheme 1:**
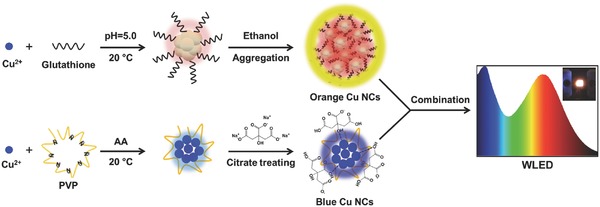
Schematics of the formation and treatment of orange and blue emitting Cu NCs, and their combination as phosphors in a down‐conversion WLED.

In this work, we demonstrate all‐copper NCs based down‐conversion WLEDs without the use of any rare‐earth phosphors or noble metal clusters. Schematics of the formation and treatment of orange and blue emitting Cu NCs, and their combination as phosphors in a down‐conversion WLED is presented in **Scheme** **1**. The orange emitting Cu NCs were prepared using GSH as both the reducing and stabilizing agent, followed by AIE treatment with ethanol. Their emission was centered around 600 nm, with a large Stokes shift and high absolute PL QYs of 24% and 43% in solution and solid state, respectively. Blue emitting Cu NCs were synthesized by reduction of PVP supported Cu(II) ions with ascorbic acid (AA), followed by surface treatment with sodium citrate. Their emission was centered around 425 nm, with the PL QY of 14% both in solution and in the solid state. WLEDs were fabricated by integrating blue and orange emitting Cu NC powders on a commercial GaN LED chip providing 370 nm excitation emission, and show favorable white light characteristics with Commission Internationale de l'Eclairage (CIE) color coordinates, CRI, and CCT of (0.36, 0.31), 92, and 4163 K, respectively. Compared with previously reported metal NC based LEDs,^9^, ^12^ a warmer white light with higher CRI is demonstrated here.

## 2 Results and Discussion

**Figure 1:**
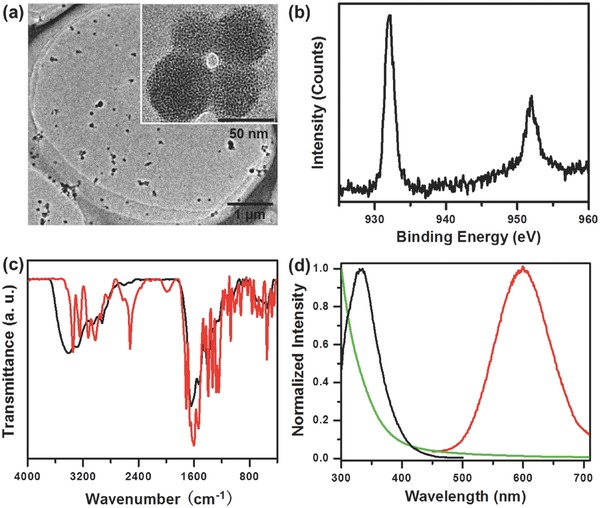
Structural and optical characterization of orange emitting Cu NCs: a) TEM image of agglomerated clusters, inset shows an enlarged view of a few quasi‐spherical agglometartes; b) XPS spectrum of Cu 2p electrons; c) FTIR spectrum of Cu NCs (red line) compared with those of GSH (black line); d) absorption spectrum (green line), PLE spectrum (black line) at detection wavelength of 600 nm, and PL spectrum (red line) at the excitation wavelength of 365 nm.

The orange Cu NCs were synthesized in water employing glutathione as both reduction agent and stabilizer, following the previously reported method,^18^ and have been injected into ethanol (see the Experimental Section for details) in order to make use of AIE effect. **Figure** **1**a shows representative transmission electron microscopy (TEM) images of orange Cu NCs, prepared with 95% volume fraction of ethanol in the solvent mixture (*f*_ethanol_ = vol_ethanol_/vol_ethanol+water_), which tend to form quasi‐spherical agglomerates with ca. 50 nm diameter, consisting of small (<2 nm) tightly aggregated nanoclusters. Two peaks at 932 and 952 eV are present in X‐ray photoelectron spectroscopy (XPS) spectrum of the sample (Figure 1b), which are assigned to 2p_1/2_ and 2p_2/3_ electrons of metallic copper.^17^ Reduction of Cu^2+^ by GSH is evidenced by the absence of any satellite peaks around 942 eV. We note that the difference between 2p_3/2_ peaks of Cu^0^ and Cu^+^ species is only 0.1 eV, so that the valence state of copper in Cu NCs may lie between zero (in the core) and one (at the surface where it is bound to sulfur), which is consent with the previous reports.^13^, ^22^ The ratio of S to Cu was estimated to be 4:1 from XPS measurements. Fourier‐transform infrared (FTIR) spectroscopy has been employed to figure out how GSH – serving as a stabilizing ligand – is anchored at the surface of Cu NCs. As shown in Figure 1c, the peak at 2524 cm^−1^ which corresponds to the S—H stretching vibration mode of GSH molecules^20^ does not appear in the FTIR spectrum of Cu NCs, indicating that GSH is connected with the Cu core through Cu—S bonding.

Figure 1d shows absorption, photoluminescence excitation (PLE), and PL spectra of orange Cu NCs. The absorption spectrum appears as rather structureless, while well resolved peak is present at 330 nm in the PLE spectrum. The shift between the PLE peak and the PL maximum centered at 600 nm is 270 nm and is an advantageous property of these Cu NCs in terms of minimizing the reabsorption of emitted light. The PL efficiency of orange Cu NCs depends on the ratio of GSH to Cu^2+^ used in the synthesis (Figure S1, Supporting Information) and on pH value of the solution (Figure S2, Supporting Information), reaching the maximum for five GSH molecules per Cu^2+^ ion, and pH equal 5.0. With the above optimized synthetic conditions, an absolute PL QY of 24% has been achieved for GSH‐capped Cu NCs in solution with *f*_ethanol_ of 95%, exceeding the emission intensity of most of the previously reported cases.^17^, ^18^

**Figure 2:**
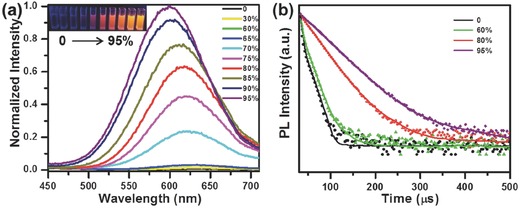
a) PL spectra (excitation wavelength 365 nm) and b) PL decay profiles (excitation wavelength 320 nm; recorded at the corresponding emission maximum) of the orange emitting Cu NCs in the water/ethanol mixture with increasing volume fraction of ethanol, *f*_ethanol_ (%). Inset in (a) shows the photographs of the respective solutions under UV light, demonstrating increasing emission intensity.

The injection of a mixture of Cu^2+^ and GSH into ethanol during the synthesis is vital to obtain strongly emitting orange Cu NCs. Clusters synthesized in pure water show only a weak red emission under UV light (**Figure** **2**a). After injection into ethanol a strong orange luminescence appears, and grows in intensity for the increasing *f*_ethanol_, accompanied by a blue shift of the emission maximum (Figure 2a), which are typical signatures of the AIE enhancement.^18^, ^20^ Ethanol, a weak polar solvent, disrupts the hydration shell of Cu NCs, leading to a charge neutralization and aggregation of clusters. Absorption spectra of Cu NCs injected into solutions with different *f*_e_ (Figure S3, Supporting Information) evidence on the different degree of scattering due to the density variation of aggregates.

To gain further insights into the mechanism of the solvent induced PL enhancement, we recorded PL decay curves of Cu NCs in water/ethanol solvent mixtures with different *f*_ethanol_ (Figure 2b), which were fitted by two to four exponential functions as summarized in **Table** **1**. From the fitting results, increasing average PL lifetimes of 1.3, 3.2, 18, and 28 μs are derived for Cu NCs synthesized in solutions with *f*_ethanol_ of 0%, 60%, 80%, and 95%, respectively. Such long (microseconds) excited state lifetimes, together with the observed large spectral shift between the PLE and PL maxima suggest that the emission of Cu clusters occurs through a phosphorescence mechanism, attributed to ligand‐to‐metal charge transfer from the sulfur atoms of GSH to the Cu atoms in the core, followed by a radiative relaxation through a metal‐centered triplet state, which is a commonly observed feature of thiolate‐capped metal NCs.^18^, ^23^ Increased degree of aggregation in solutions with increasing *f*_ethanol_ leads to a stronger inter‐ and intracluster interactions, resulting in the restriction on the molecular vibrational, rotational, and torsional movements of Cu NCs.^24^ This reduces the probability of nonradiative path and activates the radiative decay, which results in a longer PL lifetime and enhanced PL intensity.^23^, ^25^ A blue shift of PL maximum with the increase of aggregation has been previously attributed to predominance of the inter‐Cu interactions over the intra‐Cu interactions in the clusters, resulting in an increase of average Cu(I)···Cu(I) distance.^9^, ^23^, ^26^

**Table 1:** PL emission maxima (nm), PL lifetimes (*τ*_1–4_, μs), and fractions of the emission intensity (*f*_1–4_, %) obtained from the fitting of experimental PL decay data by two to four exponential functions for the orange emitting Cu NCs prepared in water/ethanol mixtures with different volume fraction of ethanol (*f*_ethanol_), together with calculated average PL lifetime *τ*_average_ (μs)

*f*_ethanol_ [%]	Emission [nm]	*τ*_1_ (*f*_1_)	*τ*_2_ (*f*_2_)	*τ*_3_ (*f*_3_)	*τ*_4_ (*f*_4_)	*τ*_average_
0	670	0.7 (92.6)	8.8 (7.4)	–	–	1.3
60	650	0.6 (51.8)	2.2 (21.4)	8.5 (26.1)	29.9 (0.7)	3.2
80	640	2.6 (6.5)	3.1 (6.7)	19.4 (82.8)	42.2 (4.0)	18.0
95	610	2.5 (2.9)	2.6 (3.7)	29.1 (92.2)	86.8 (1.2)	28.0

**Figure 3:**
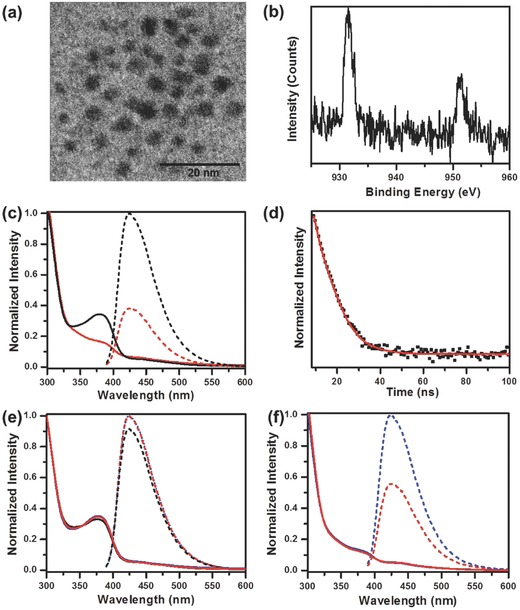
Structural and optical characterization of blue emitting Cu NCs: a) TEM image; b) XPS spectrum of Cu 2p electrons; c) absorption (solid lines) and PL (dashed lines, excitation wavelength 380 nm) spectra of the samples before (red) and after (black) citrate treatment; d) PL decay profile (excitation at 320 nm, recorded at emission peak of 420 nm); e) absorption (solid lines) and PL (dashed line) spectra taken on the citrate treated sample after different storage time (blue line – 0 d, red line – one week; black line – one month); f) absorption (solid lines) and PL (dashed line) taken on the sample without citrate treatment after different storage time (blue line – 0 d, red line – one week).

The synthesis of blue Cu NCs was carried similar to our previous report,^21^ through reduction of PVP supported Cu^2+^ ions by a mild reducing agent AA. Quasi‐spherical clusters with an average diameter of 3 nm are observed in TEM images (**Figure** **3**a). The oxidation states of copper in the clusters has been studied by XPS measurements (Figure 3b), with the findings similar to the presented above for the orange emitting Cu NCs. The postpreparative sodium citrate treatment results in almost threefold increase of the PL intensity of NCs, without any change of their PL spectral profile and PL peak position (Figure 3c). An increase of the peak intensity at 380 nm was also recorded in the absorption spectrum (Figure 3c), which is attributed to the enhanced interaction between Cu atoms and the electron rich oxygen atoms of citrate, similar to our previous findings on the surface treatment of Cu NCs with sulfur containing ligands.^27^ Both concentration of citrate (Figure S4, Supporting Information) and pH of the system (Figure S5, Supporting Information) influence the degree of the PL enhancement for Cu NCs, with optimal results achieved for 10 × 10^−3^ m sodium citrate at pH 4.5, when PL QY reached 14%. The PL decay curve of blue Cu NCs is shown in Figure 3d. It can be fitted by a three‐exponential function, with three components of 1.0 ns (38.1%), 3.2 ns (56.6%), and 8.0 ns (5.3%) providing an average PL lifetime of 2.6 ns. Different to the orange emitting Cu NCs with PL lifetime in the microsecond range, such short (nanoseconds) PL lifetime is attributed to the emission from the singlet excited state, as has been previously observed for some Cu NCs.^18^, ^28^

The PL stability of metal NCs is a key concern for their practical applications.^29^, ^30^ Previously reported blue emitting Cu NCs often suffered poor stability in solution, caused by an easy oxidation of small Cu particles. We found out that PL of blue emitting Cu NCs treated by citrate is very stable in solution at ambient conditions. Figure 3e presents absorption and PL spectra of Cu NCs after storage for different time intervals: very marginal changes were observed (<10% decrease in PL and stable absorption intensity) even after one month storage. This is in strong contrast to the nontreated Cu NCs whose PL intensity decreased by 50% after one week of storage under ambient conditions (Figure 3f). Citrate treatment apparently provides an efficient protection shell diminishing the possibility of the diffusion of oxidative species toward the Cu core, which is in good correlation with previous report on the enhanced stability of metal NCs through proper ligand‐shell engineering.^30^

**Figure 4:**
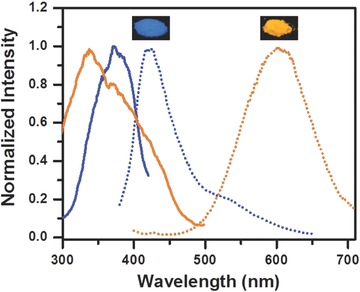
Optical characterization of NC powders (shown as insets under UV illumination). PLE spectra of blue Cu NCs (solid blue line, detection wavelength 460 nm) and orange Cu NCs (solid orange line, detection wavelength 580 nm), as well as PL spectra of blue Cu NCs (dotted blue line) and orange Cu NCs (dotted orange line), both excited at 365 nm.

Transfer from a solution to the powdered state is a common step to obtain phosphors for the further use in LED applications. During this process, a decrease or even total quenching of emission could happen for metal NCs, caused by aggregation and/or oxidation or both,^30^ while this was not the case for Cu NCs reported here. The powder of the blue emitting Cu NCs has been obtained by drying the respective solution in vacuum oven at 50 °C, while the powder of the orange emitting NCs was obtained by drying of centrifuged precipitate. **Figure** **4** presents photographs of bright emitting powders taken under UV illumination, together with the PLE and PL spectra of the respective solid state samples. There was no change in the position of emission maximum for blue Cu NCs in the solution and in powder, and their PL QYs were estimated as 14% in the both form. This can be attributed to the protective role of PVP providing steric hindrance to isolate and stabilize the Cu NCs both in solution and in powdered state. For orange Cu NCs, the PL maximum shifted to the red by 15 nm upon transferring them from solution to the powder, while the PL QY increased even further, from 24% to 43%. The strong PL enhancement in the solid state further supports the AIE mechanism.

**Figure 5:**
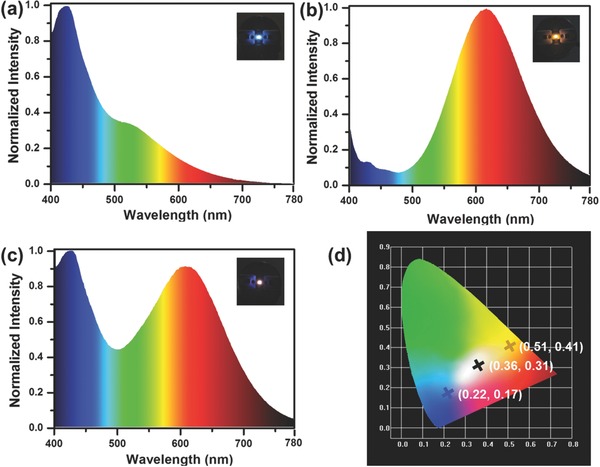
Emission spectra of monochrome down‐conversion LEDs fabricated by using a) blue emitting Cu NCs and b) orange emitting Cu NCs. c) An emission spectrum of the WLED fabricated by combination of these two kinds of Cu NCs. Insets in (a), (b), and (c) provide photographs of operating blue, orange, and white LEDs, while d) shows CIE coordinates of three respective LEDs.

PL spectra of blue and orange Cu NCs shown in Figure 4 evidence on the fact that the emission of their mixture covers the whole visible spectral range from 400 to 700 nm, thus being advantageous for generating white light. Moreover, there is a favorable overlap of their PLE spectra in the 300–400 nm spectral range, allowing us to employ a single excitation UV source to generate white light. The high PL QY and a large Stokes shift of orange Cu NCs are useful properties to achieve warm light with high CRI and color stability. Owing to these advantageous optical properties, we have employed the blue and orange Cu NCs as color conversion phosphors for down‐conversion monochrome and white LEDs. Commercial GaN chips providing 370 nm emission (spectrum shown in Figure S6, Supporting Information) was used as a light source for all fabricated devices. Emission spectra and photographs of operating monochrome LEDs are shown in **Figure** **5**a,b; they generate blue and orange light with the color coordinates (0.22, 0.17) and (0.51, 0.41), respectively (Figure 5d).

WLED have been fabricated by employing two‐component mixtures of blue and orange emitting Cu NCs, with an emission spectrum shown in Figure 5c. The WLEDs emit white light with CIE, CRI, and CCT of (0.36, 0.31), 92, and 4163 K, respectively. Table S1 (Supporting Information) compares the performance of our devices with those of previously reported WLEDs employing metal NCs.^9^, ^12^, ^21^ Higher CRI and lower CCT have been achieved, attributed to the full color coverage of these two phosphors, high PL efficiency, and a large Stokes shift of orange Cu NCs. To examine optical stability of WLEDs, their CIE, CRI, and CCT were measured at different working currents. As shown in **Table** **2**, with the increasing of current from 20 to 120 mA, a slight change of CIE from (0.358, 0.307) to (0.363, 0.310) is observed, which is very close to the ideal CIE for pure white emission (0.33, 0.33). Slight variation of CCT (less than 5%) under the current changing from 20 to 120 mA has also been recorded. Such minor change of CIE and CCT has little influence on the CRI, which exceeds 92 for all current. This is related to minor changes of the emission spectra of WLEDs under different working current, with almost the same peak positions and intensity ratio of blue and orange Cu NCs (Figure S7, Supporting Information) due to the large Stokes shift of orange Cu NCs and the minor overlap between the emission of blue Cu NCs and the absorption of orange Cu NCs in our LEDs employing a fivefold excess of the former. The luminous efficiencies of WLEDs under different working currents were recorded and shown in Figure S8 (Supporting Information). There is a linear relationship between the light luminance and the currents (*R* = 0.991), which suggests that Cu NCs do not show excitation intensity dependent quantum yield.

**Table 2:** CIE, CCT, and CRI of all‐copper based WLEDs operating at different working currents

Current [mA]	Parameters		
	CIE (*x*, *y*)	CCT [K]	CRI
20	(0.358, 0.307)	4163	92.2
40	(0.363, 0.310)	3953	92.1
80	(0.361, 0.308)	4021	92.1
100	(0.360, 0.307)	4046	92.1
120	(0.360, 0.306)	4053	92.1

## 3 Conclusions

WLEDs employing solely Cu NCs as phosphors are demonstrated for the first time. Orange emitting Cu NCs are synthesized by employing GSH as both reducing and stabilizer agent, through injection of precursors into the water/ethanol mixtures to make use of the AIE enhancement effect. Their emission is centered around 600 nm, with a large Stokes shift and high absolute PL QYs of 24% and 43% in solution and solid state, respectively. Optical studies indicate that the PL of orange Cu NCs can be assigned to triplet excited states, and their solvent induced aggregation restricts nonradiative decays, concominant with the enhanced emission efficiency. Blue Cu NCs are synthesized by an AA reduction of copper ions supported on a PVP backbone. Postpreparative sodium citrate treatment improves both their PL efficiency and stability, resulting in samples emitting at 425 nm, with the PL QY of 14% both in solution and in the solid state. Blue and orange emitting Cu NCs were processed into powders and employed as phosphors of both monochrome and white down‐conversion LEDs which are rare‐earth free and nonexpensive. A white light achieved shows favorable characteristics with CIE, CRI, and CCT of (0.36, 0.31), 92, and 4163 K. Results of our study provide a convenient way to improve the PL QY and the stability of Cu NCs, which may intensify further efforts toward the employment of copper NCs in WLED applications.

## 4 Experimental Section

*Materials*: All chemicals including copper(II) nitrate (Cu(NO_3_)_2_), polyvinylpyrrolidone (PVP, average molecular weight: 40 000), ascorbic acid (AA), sodium citrate, and glutathione (GSH) were purchased from Sigma‐Aldrich, USA. Components of silicone resin OE‐6551A and OE‐6551B were purchased from Dow Corning Co. Milli‐Q grade deionized water (18.2 MΩ cm) was used for all experiments.

*Synthesis of Orange Emitting Cu NCs*: 0.5 mL of 50 × 10^−3^ m aqueous solution of Cu(NO_3_)_2_ and 2.5 mL of 50 × 10^−3^ m aqueous solution of GSH were mixed with 2.0 mL of water and pH was adjusted to 5.0 using 2 m NaOH. A milky suspension was initially formed (at pH 2.7), turning to light yellow, transparent solution at pH higher than 3.7. In order to make use of the AIE effect, specific amount of this solution was injected into ethanol to prepare mixtures with different volume fractions of ethanol varying from 30 to 95. Powdered samples of Cu NCs were obtained after drying the precipitate (obtained by centrifugation at 14 000 rpm) under vacuum at 50 °C.

*Synthesis of Blue Emitting Cu NCs*: 0.5 g PVP was dissolved in 10 mL of water under ultrasound treatment for 10 min. pH of the PVP solution was adjusted to 6.0 using 1 m NaOH, and 0.1 mL of Cu(NO_3_)_2_ (0.1 m in water) and 1 mL AA (0.1 m in water) were added. After 6 d reaction at room temperature, the obtained solution was dialyzed against water through a membrane with a molecular weight cutoff of 14 000 for 48 h. 0.5 mL of 0.1 m aqueous solution of sodium citrate was mixed with 4.5 mL of Cu NC solution taken from the dialysis tube, and pH was adjusted to 4.5 using 1 m HCl. After 5 d incubation at room temperature, the mixture was dialyzed against water for another 48 h, and the NC solution inside the dialysis tube was used for further characterization and measurements. Powdered samples of Cu NCs were obtained after drying the solution under vacuum at 50 °C.

*Fabrication of LEDs*: For the fabrication of monochrome blue and orange LEDs, 0.1 g of blue or orange Cu NC powder was mixed with 0.1 g of thermal‐curable silicone resin OE‐6551A. For the fabrication of WLEDs, 0.1 g of blue Cu NC powder and 20 mg of orange Cu NC powder were mixed with 0.1 g of OE‐6551A. Mixtures were dried under 50 °C for 1 h, and mixed with 0.2 g of the hardener OE‐6551B. The devices were constructed by applying the above mixtures onto commercially available GaN LED chips of surface mounted device type, with the peak emission wavelength centered at 370 nm (EPILED Co., Ltd).

*Characterization*: Varian Cary 50 UV–visible spectrophotometer and Varian Cary Eclipse fluorescence spectrometer were employed to record the absorption and PL spectra, respectively. Time‐resolved PL lifetime measurements were carried out using a time‐correlated single‐photon counting setup with a 320 nm laser as the excitation light source. The PL QY, defined as the ratio between photons emitted and absorbed by the sample, was determined by an absolute method using an integrating sphere (Edinburgh Instruments) with its inner surface coated with BENFLEC, attached to the spectrofluorimeter FLS920P (Edinburgh Instruments). Spectral correction curves for the sphere and emission detectors were provided by Edinburgh Instrument. Excitation wavelength for PL QY measurements was 375 nm. TEM images were obtained on a Philips CM 20 microscope operating at 200 kV. XPS has been performed on an ESCALAB‐MKII 250 photoelectron spectrometer (Thermo, USA). FTIR spectra were recorded on a Perkin‐Elmer Spectrum 100 FTIR spectrometer. Luminous efficiency, CIE color coordinates, CCT, and CRI of LEDs were measured under different forward currents in an integrating sphere with a high accuracy array rapid spectroradiometer (Haas‐2000, Everfine Co., Ltd, China).

## Supporting information

As a service to our authors and readers, this journal provides supporting information supplied by the authors. Such materials are peer reviewed and may be re‐organized for online delivery, but are not copy‐edited or typeset. Technical support issues arising from supporting information (other than missing files) should be addressed to the authors.

Supplementary
