# Supplementary material for: All‐Copper Nanocluster Based Down‐Conversion White Light‐Emitting Devices
Source: Adv Sci (Weinh). 2016 Jun 21;3(11):1600182. doi: 10.1002/advs.201600182 (PMC5102667; doi:10.1002/advs.201600182)
Supplement: Supplementary file 1 — Supplementary [file ADVS-3-0c-s001.pdf]

Copyright WILEY-VCH Verlag GmbH & Co. KGaA, 69469 Weinheim, Germany, 2016.

ADVANCED SCIENCE
Open Access

Supporting Information

for *Adv. Sci.,* DOI: 10.1002/advs.201600182

All-Copper Nanocluster Based Down-Conversion White Light-Emitting Devices

*Zhenguang Wang, Bingkun Chen, Andrei S. Susha, Weihua Wang, Claas J. Reckmeier, Rui Chen, Haizheng Zhong, and Andrey L. Rogach**

SUPPORTING INFORMATION

All-copper nanocluster based down-conversion white light-emitting devices

Zhenguang Wang[1], *Bingkun Chen*[1,2], *Andrei S. Susha*[1], *Weihua Wang*[1], *Claas Reckmeier*[1], *Rui Chen*[1], *Haizheng Zhong*[2], *and Andrey L. Rogach*[1,*]

[1] Department of Physics and Materials Science and Centre for Functional Photonics (CFP), City University of Hong Kong, 83 Tat Chee Avenue, Kowloon, Hong Kong SAR.

[2] Beijing Key Laboratory of Nanophotonics and Ultrafine Optoelectronic Systems, School of Materials Science & Engineering, Beijing Institute of Technology, Beijing, 100081, China.

* Corresponding author. E-mail: andrey.rogach@cityu.edu.hk

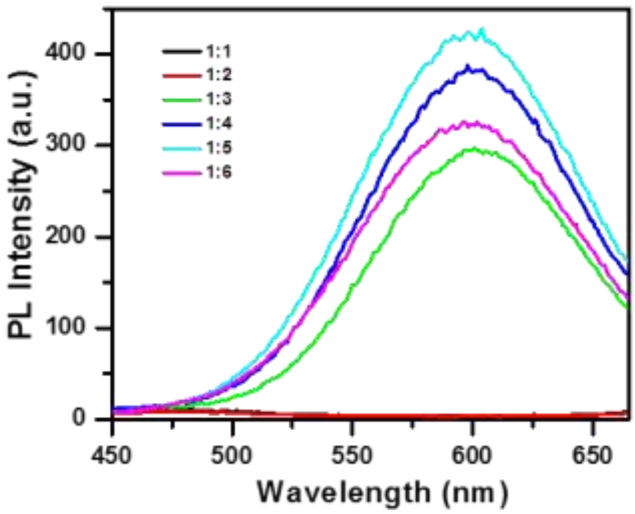

Figure S1. PL spectra (excited at 350 nm) of orange Cu NCs synthesized under different ratios of Cu^{2+} to GSH as indicated on the frame.

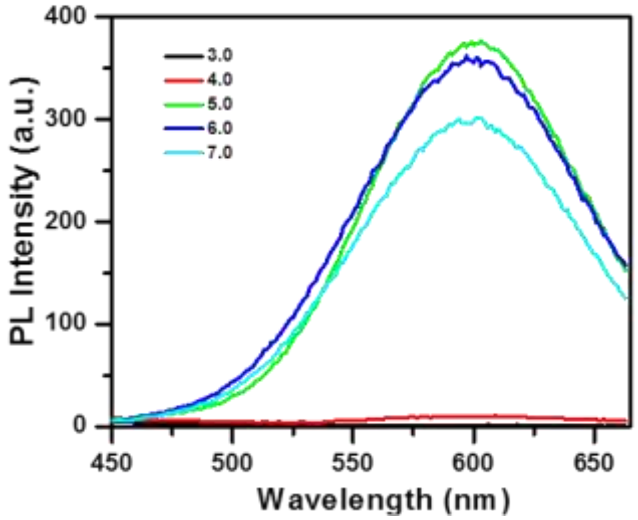

Figure S2. PL spectra (excited at 350 nm) of orange Cu NCs synthesized under different pH as indicated on the frame.

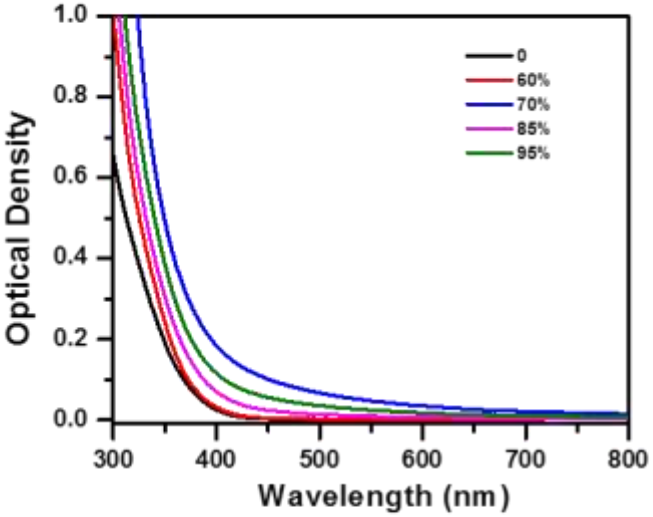

Figure S3. Absorption spectra of Cu^{2+} / GSH mixtures injected into ethanol for different values of $f_{ethanol}$ indicated on the frame.

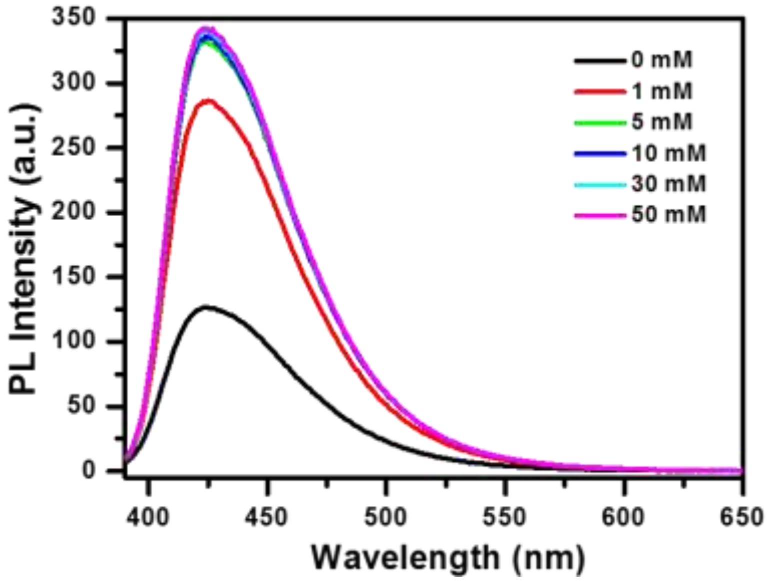

Figure S4. PL spectra (excited at 380nm) of blue Cu NCs treated by different amounts of sodium citrate concentration as indicated on the frame.

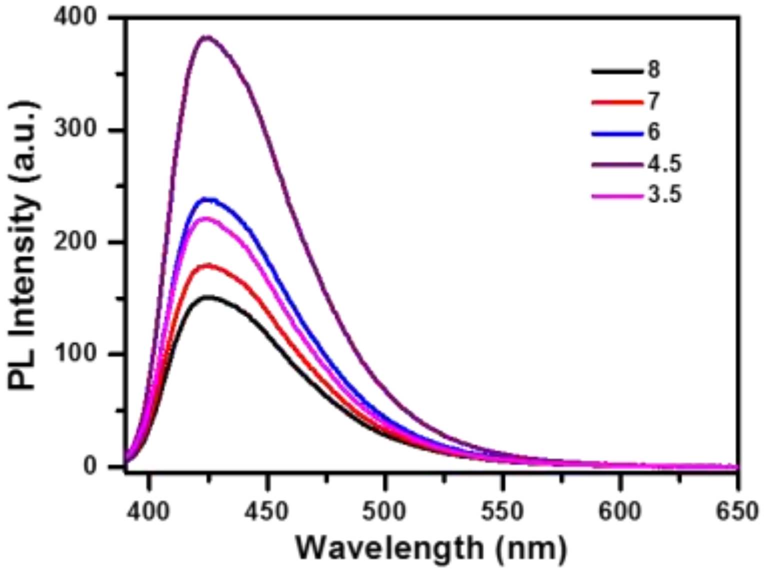

Figure S5. PL spectra (excited at 380nm) of blue Cu NCs synthesized under different pH as indicated on the frame.

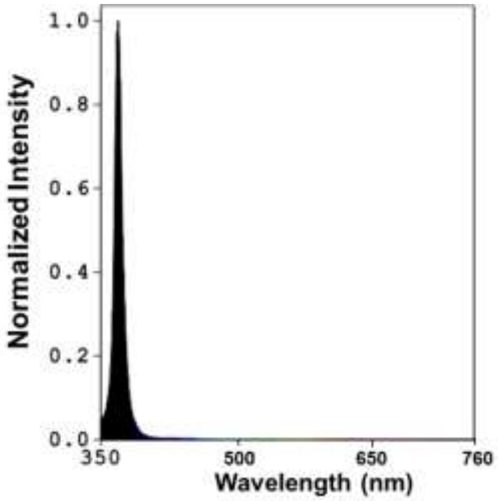

Figure S6. Emission spectrum of GaN LED chip used for fabrication of down-conversion LEDs.

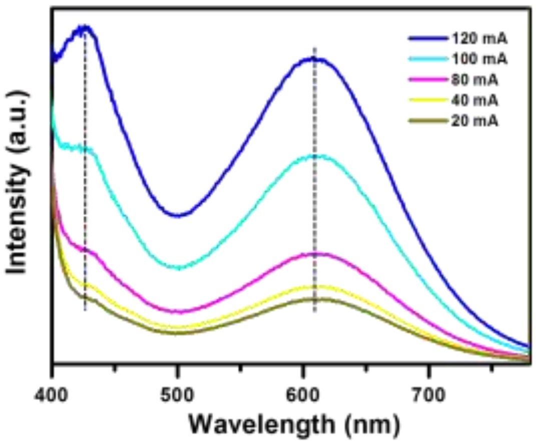

Figure S7. Emission spectra of all-copper based down conversion WLEDs operating under different working currents.

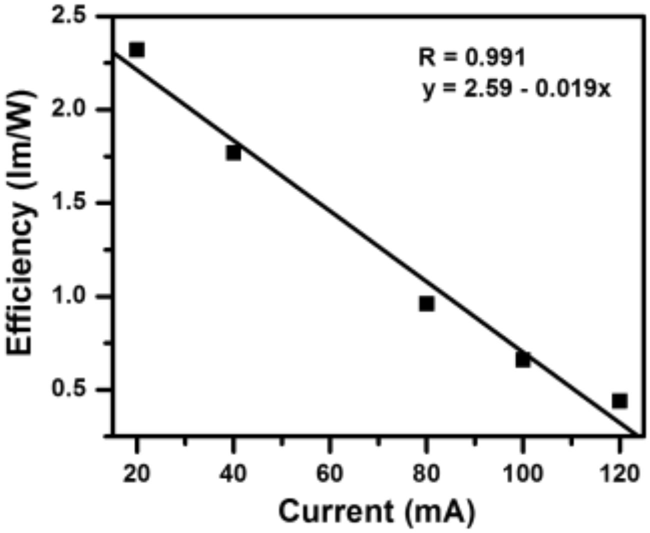

Figure S8. Relationship between the working current of all-copper based down conversion WLEDs and their luminous efficiency.

Table S1. Performance comparison of all-copper based down-conversion WLEDs with previously reported WLEDs employing metal nanoclusters

Reference	CIE(x, y)	CCT (K)	CRI
1	(0.32, 0.36)	/	/
2	(0.31, 0.36)	6577	88
3	(0.35, 0.33)	4742	92
This work	(0.36, 0.31)	4163	91

References

1. Z. Wu, J. Liu, Y. Gao, H. Liu, T. Li, H. Zou, Z. Wang, K. Zhang, Y. Wang, H. Zhang and B. Yang, *J. Am. Chem. Soc.*, 2015, **137,** 12906.

2. J. Liu, Z. Wu, T. Li, D. Zhou, K. Zhang, Y. Sheng, J. Cui, H. Zhang and B. Yang, *Nanoscale* 2016, **8,** 395.

3. Z. Wang, A. Susha, B. Chen, C. Reckmeier, O. Tomanec, R. Zboril, H. Zhong, A. Rogach, *Nanoscale* 2016, **8**, 7197.
